# Research Progress and Prospects on Poisoning Mechanism and Anti-Poisoning Modification of Cerium-Based NH_3_-SCR Denitrification Catalysts

**DOI:** 10.3390/ma19153223

**Published:** 2026-07-28

**Authors:** Qi Zhao, Zhuoya Qu, Suqian Gu, Shengli An, Shan Ren, Yifan Chai

**Affiliations:** 1 School of Rare Earth Industry (School of Rare Earth Engineering and Technology), Inner Mongolia University of Science and Technology, Baotou 014010, China; zq15391133704@163.com (Q.Z.); 15635222600@163.com (Z.Q.); shengli_an@126.com (S.A.); 2 Engineering Research Center of Carbon Neutrality for Universities of Inner Mongolia Autonomous Region, Baotou 014010, China; 3 School of Energy and Power Engineering, Xi’an Jiaotong University, Xi’an 710049, China; shan.ren@cqu.edu.cn

**Keywords:** cerium-based catalysts, NH_3_-SCR, poisoning mechanism, doping modification, anti-poisoning performance, rare earth catalysis

## Abstract

**Highlights:**

**What are the main findings?**

- The poisoning and deactivation mechanisms of Ce-based NH_3_-SCR catalysts exposed to industrial sintering flue gas are systematically categorized and elaborated.
- Element doping strategies for boosting the anti-SO_2_, anti-heavy metal and anti-water tolerance of CeO_2_ catalysts are comprehensively summarized and contrasted.

**What are the implications of the main findings?**

- The intrinsic rules of electronic structure and oxygen vacancy regulation for anti-poisoning performance are clarified to guide catalyst structural design.
- Prospects are proposed for the industrialized application of robust Ce-based denitrification materials under complex flue gas conditions.

**Abstract:**

Ammonia selective catalytic reduction (NH_3_-SCR) has become the mainstream core technology for denitrification of industrial sintering flue gas, owing to its high denitrification efficiency and mild reaction conditions. Cerium-based catalysts, with CeO_2_ as the primary component, have been identified as a promising system for replacing traditional vanadium-based and noble metal catalysts. These catalysts rely on the reversible Ce^3+^/Ce^4+^ redox cycle and exhibit favorable oxygen storage-release capacity derived from lattice oxygen migration. However, the presence of multiple impurities in industrial flue gas can lead to catalyst poisoning and restrict its industrial application. Consequently, there is an urgent need for the development of cerium-based catalysts that exhibit both high denitrification activity and excellent resistance to sulfur, heavy metal and water poisoning for the engineering application of NH_3_-SCR technology. The present paper undertakes a systematic analysis of the poisoning mechanisms of various pollutants on cerium-based SCR denitrification catalysts. In addition, it discusses the enhancement effects of doping modification with rare earth elements, transition metal elements and non-metallic elements on the sulfur resistance of catalysts. Furthermore, it reveals the intrinsic laws of different modification pathways in improving sulfur resistance by optimizing the electronic structure, regulating surface acidic sites and inducing the formation of oxygen vacancies. The present study provides theoretical support for the design and industrial application of anti-poisoning cerium-based catalysts.

## 1. Introduction

Nitrogen oxides (NO*_x_*) are conventional air pollutants that pose a significant threat to the ecological environment and human health [1,2], with industrial emissions representing their primary source. Specifically, the NO*_x_* concentration in iron and steel sintering flue gas reaches 350–450 mg/m^3^, characterized by high-emission intensity and significant control difficulty [3,4]. In order to effectively regulate NO*_x_* pollution, major economies worldwide have formulated and implemented stringent emission control standards. China’s ultra-low emission standards for the iron and steel industry and pelletizing industry (GB 28662-2012) stipulate the NO*_x_* emission limit for iron and steel sintering flue gas as 50 mg/m^3^ [5], with certain key regions implementing a further reduction to 35 mg/m^3^ [6].

These standards have driven the upgrading of denitrification technologies towards low energy consumption, a wide-temperature window, strong anti-poisoning ability, and high stability. Among these technologies, ammonia selective catalytic reduction (NH_3_-SCR) has become the mainstream technology due to its high removal efficiency (>90%), excellent N_2_ selectivity and stable operation. The intrinsic performance and anti-poisoning ability of denitrification catalysts have a direct impact on the engineering application effect of NH_3_-SCR. These catalysts are pivotal in overcoming denitrification bottlenecks under complex conditions [7,8,9,10].

At present, the majority of commercial denitrification catalysts are of the vanadium-based and noble metal-based systems. Vanadium-based catalysts (predominantly composed of V_2_O_5_-WO_3_/TiO_2_ systems) have established a predominant presence within the field due to their well-established preparation methods, a broad applicable temperature range (300–400 °C), and consistent performance [11,12]. However, it should be noted that vanadium is toxic and can lead to secondary pollution [13,14]. Additionally, their low-temperature activity (<250 °C) is inadequate for the wet desulphurised flue gas process. Noble metal catalysts (e.g., Pt, Pd-based) exhibit excellent activity at low temperatures (100–200 °C) and dust resistance [15,16]. However, the scarcity of noble metal resources results in high costs. Furthermore, these systems are vulnerable to sulfur poisoning when exposed to sulfur-containing conditions over extended periods. Additionally, the oxidation of NH_3_ is likely to occur at elevated temperatures, resulting in a reduction in N_2_ selectivity, thereby hindering the fulfillment of the criteria necessary for large-scale industrial applications [17].

In comparison with conventional catalysts, cerium-based catalysts represent optimal alternatives due to their distinctive advantages. The reversible conversion of Ce^3+^/Ce^4+^ valences confers exceptional redox properties, thereby activating reaction intermediates. The distinctive lattice oxygen migration capability of CeO_2_ provides substantial oxygen storage and release capacity, thereby compensating for oxygen concentration fluctuations [18]. The abundance of surface acid sites offers active centers for NH_3_ adsorption and activation [19]. It is important to note that these materials are non-toxic and environmentally friendly, and are regarded as the most promising green substitute for vanadium-based catalysts [20,21,22].

These advantages have been systematically validated and supported by decades of pioneering work from international research teams, particularly those in Europe and the United States. The seminal review by American scholars Farrauto and Heck was the first to systematically elucidate the dominant role of the Ce^3+^/Ce^4+^ redox cycle, and quantitatively characterized the oxygen storage and release capacity (OSC) of pure ceria, laying the theoretical foundation for the core performance of ceria-based catalysts [23]. Subsequent experimental work by the Italian team led by Sartoretti et al. further confirmed the critical regulatory role of surface acid sites in the flue gas purification activity of ceria-based catalysts, providing experimental support for the optimization of acid site properties [24]. Together, these two European and American studies collectively demonstrate the unique advantages of ceria-based catalysts as green alternatives to vanadium-based catalysts.

Based on the excellent catalytic performance and promising application prospects of ceria-based catalysts, current studies on the optimization and modification of ceria catalytic systems at home and abroad mainly fall into two categories. On the one hand, composite modification with transition metals, including manganese, iron and copper, is applied to optimize ceria systems, so as to enhance catalytic activity and low-temperature adaptability. This strategy has been validated by a large number of studies [25,26,27,28,29]. On the other hand, researchers focus on the development of novel functional derivative catalytic systems. A variety of new structural catalysts have been successively developed, including metal-organic framework (MOF)-derived catalysts [30], layered double hydroxide (LDH)-derived catalysts [31], and zeolite-supported catalysts [32,33], which effectively break the structural and performance limitations of traditional ceria-based materials.

International research teams have also conducted extensive exploratory research on such novel functional derivative catalytic systems. Reviews by international research teams have systematically demonstrated that layered double hydroxide (LDH)-derived catalytic materials, with their unique tunable layered structure and abundant surface-active sites, provide an effective platform for the functional modification of ceria-based catalysts. Modified LDH-based composites exhibit significantly enhanced catalytic oxidation activity and cycling stability for pollutant removal, offering important references for the development of ceria-based catalysts for flue gas purification [34]. Studies by international research teams have further confirmed that the pore confinement effect of zeolite-based molecular sieves can effectively inhibit the sintering and agglomeration of metal active components under medium and high temperature conditions, providing a key strategy to improve the thermal stability and long-term operational performance of ceria-based catalysts [35]. In addition, existing studies reveal that multi-element doping with zirconium, tin, samarium and other elements, combined with interface regulation, can produce synergistic effects and further improve the operating condition adaptability, catalytic activity and long-term operational stability of ceria-based catalysts [36,37,38,39,40].

Nevertheless, cerium-based catalysts still suffer from serious deactivation, with NO*_x_* conversion decreasing by more than 30% in industrial flue gas, a phenomenon that is considerably more intricate than that observed in laboratory systems [41]. It has been demonstrated that elevated concentrations of SO_2_, dust, water vapor and trace heavy metals, including Zn, Pb, K and Cd, can readily result in the rapid deactivation of cerium-based catalysts [42,43,44,45,46,47]. Specifically, SO_2_ reacts with Ce to form Ce_2_(SO_4_)_3_, directly destroying the active sites of the catalyst, or forms NH_4_HSO_4_ with NH_3_ and H_2_O, causing pore blockage of the catalyst [48,49,50]. Furthermore, heavy metals occupy the active centers of the catalyst and destroy Ce-O chemical bonds, resulting in irreversible deactivation of the catalyst [51,52]. In addition, alkali metals significantly inhibit the adsorption and activation of NH_3_, and the synergistic effect of multiple pollutants further aggravates catalyst performance degradation, severely restricting its catalytic stability [53]. These deactivation problems have become the core bottleneck hindering the transition of cerium-based catalysts from laboratory research to large-scale industrial application, and also a critical issue urgently to be solved in the current research field of cerium-based denitrification catalysts.

In view of this, the present paper focuses on the anti-poisoning research of cerium-based catalysts under complex industrial flue gas conditions. The paper systematically sorts out their main poisoning types and core poisoning mechanisms, comprehensively summarizes existing anti-poisoning modification strategies, and compares the technical principles, anti-poisoning effects and applicable scenarios of different strategies. This review first clarifies the multi-pollutant synergistic poisoning mechanism at the electronic level and proposes a multi-element synergistic modification route for industrial flue gas. The objective of this study is to provide a theoretical foundation for the targeted design and precise construction of high-performance anti-poisoning cerium-based denitrification catalysts. The study also aims to promote their engineering application in the field of ultra-low emission of industrial flue gas, such as that produced by iron and steel sintering.

## 2. Characteristics of Complex Industrial Flue Gases and Overview of Multi-Pollutant Synergistic Poisoning

As the primary technology for industrial flue gas denitrification, NH_3_-SCR catalysts are inevitably subjected to harsh operating conditions, where catalytic stability is highly governed by the complexity of flue gas compositions. In contrast to the idealized single-component or low-pollutant environments commonly adopted in fundamental studies, real-world industrial flue gases—particularly those from iron and steel sintering—represent a typical multi-pollutant system coexisting with NO*_x_*, SO_2_, H_2_O, and dust, alongside various heavy metals, alkali/alkaline earth metals, and trace corrosive components such as HCl and phosphorus-containing species. The mutual interactions and simultaneous presence of these impurities constitute the primary source of rapid deactivation observed in cerium-based catalysts under practical conditions [54,55,56,57,58].

**Figure 1:**
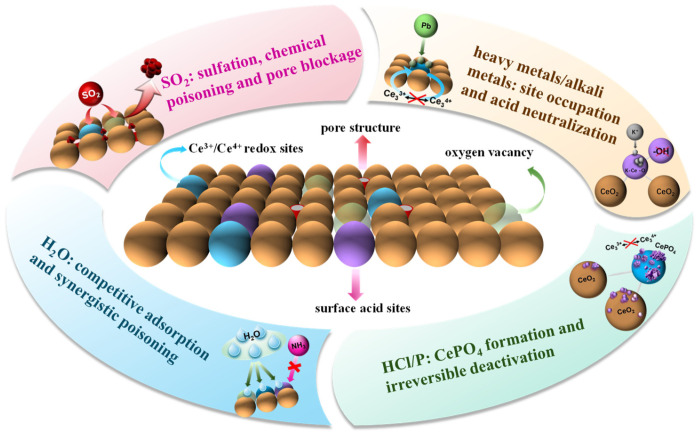
Schematic diagram of synergistic poisoning mechanism of CeO_2_-based ammonia selective catalytic reduction (NH_3_-SCR) catalysts. Brown spheres represent the CeO_2_ substrate; blue spheres are Ce^3+^/Ce^4+^ redox sites, and purple spheres are surface acid sites; translucent green spheres stand for oxygen vacancies; red hollow channels represent pore structures. Red crosses indicate the irreversible destruction of the Ce^3+^/Ce^4+^ redox cycle. The peripheral arrows illustrate the diffusion and poisoning pathways of various flue gas toxicants (sulfur dioxide, heavy metals/alkali metals, water, hydrogen chloride/phosphorus) on the catalyst surface.

Figure 1 systematically illustrates the synergistic poisoning mechanism induced by major flue gas pollutants over CeO_2_-based NH_3_-SCR catalysts. Centered on the CeO_2_ catalyst matrix, the catalyst relies on three core functional sites to drive SCR reactions: Ce^3+^/Ce^4+^ redox active sites, surface Lewis and Brønsted acid sites, and lattice oxygen vacancies. Meanwhile, stacked particles form interconnected pore channels for gas diffusion. In detail, each type of pollutant targets specific functional sites and structures step by step: SO_2_ molecules first adsorb on Ce active sites and then trigger sulfation reactions to form stable metal sulfates, accompanied by physical pore blockage; heavy metal ions migrate along the catalyst surface and permanently occupy core redox sites, further breaking Ce-O chemical bonds inside the crystal lattice; alkali metal cations preferentially bind to acidic sites and neutralize their activity; H_2_O molecules compete for adsorption sites and amplify the toxicity of other pollutants through auxiliary reactions; HCl and phosphorus species react with cerium components to generate thermodynamically stable inorganic salts, leading to irreversible crystal damage. Collectively, the superimposition of these independent poisoning behaviors produces an obvious 1 + 1 > 2 synergistic effect, which gradually destroys the catalyst’s functional sites and pore structure, and ultimately causes severe performance degradation.

The unique characteristics of complex industrial flue gases impose three distinct challenges for cerium-based catalysts, which are substantially different from ideal laboratory conditions. First, the flue gas temperature is predominantly confined to the medium-low temperature range of 100–400 °C, which limits the full activation of SCR reactions. Second, the multi-pollutant synergy accelerates the deactivation rate, thereby significantly shortening the catalyst lifespan. Third, large fluctuations in flue gas composition impose stringent requirements on catalyst structural stability and anti-interference capability. Under such realistic operating environments, the development of robust cerium-based catalysts with superior anti-poisoning resistance becomes increasingly imperative.

Therefore, clarifying the comprehensive features of industrial flue gases and deciphering the multi-pollutant synergistic poisoning behavior are not only essential for understanding the deactivation mechanisms of CeO_2_-based catalysts but also provide crucial theoretical guidance for the design of targeted modification strategies, such as surface doping, structure optimization, and anti-poisoning encapsulation, for future industrial applications.

## 3. Denitrification Deactivation Mechanism of Cerium-Based Catalysts in Industrial Flue Gases

Cerium-based catalysts are considered ideal alternative systems for low-temperature denitrification in complex industrial flue gases due to their excellent low-temperature redox performance, tunable oxygen vacancies, and good potential resistance to alkali metals/heavy metals. However, under the above-mentioned real flue gas conditions (medium-low temperature, multi-component, and strong fluctuation), they still suffer severe deactivation. Different from vanadium-based catalysts, whose deactivation is mainly caused by surface deposition, cerium-based catalysts experience combined damage of chemical bond fracture, electronic structure change and physical structure collapse, and their deactivation mechanisms are more complex. For iron and steel sintering flue gas, the deactivation mechanisms of cerium-based catalysts can be mainly summarized as follows.

### 3.1. SO_2_-Induced Denitrification Deactivation Mechanism

SO_2_ is the most dominant toxic component in industrial flue gas, and its deactivation effect on cerium-based catalysts follows a dual mode of chemical poisoning and physical blockage. The whole failure process can be divided into continuous stages, and it is also regulated by flue gas temperature and water vapor content.

#### 3.1.1. Chemical Poisoning

Chemical poisoning is the core pathway: SO_2_ undergoes irreversible chemical reactions with surface-active components to generate thermally stable metal sulfates (e.g., Ce_2_(SO_4_)_3_, MnSO_4_ from Ce, Mn). This causes three negative effects: permanently occupying surface-active sites and directly blocking the adsorption channels of NO*_x_* and NH_3_; destroying Ce^3+^/Ce^4+^ redox balance, reducing surface oxygen vacancies and impairing redox capacity, thus inhibiting NO*_x_* oxidation and NH_3_ activation; covering Lewis and Brønsted acid sites, damaging acid site quantity and strength distribution, and interrupting core SCR denitrification steps [35,59,60,61,62,63,64].

#### 3.1.2. Physical Blockage

Physical blocking mainly occurs below 300 °C, resulting from the synergy of SO_2_, NH_3_ and H_2_O, which react to form condensable ammonium salts (NH_4_HSO_4_ and (NH_4_)_2_SO_4_) with low melting points (150–200 °C). These salts condense and deposit on the catalyst surface: blocking pore structure, reducing specific surface area and pore size to hinder reactant diffusion; covering active sites, superimposing with chemical poisoning to accelerate deactivation [65,66,67,68].

**Figure 2:**
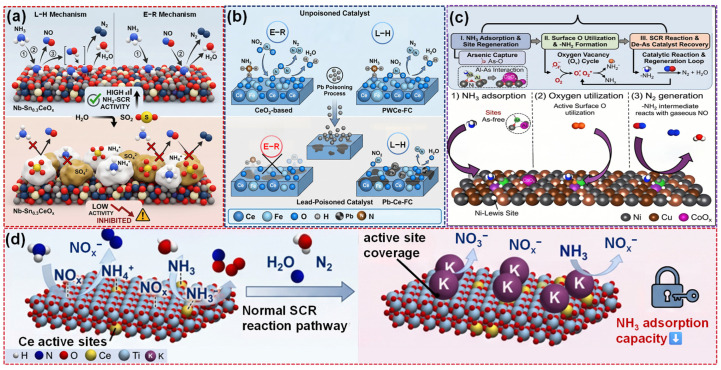
(**a**) schematic diagram of classic L-H and E-R reaction mechanisms for NH_3_-SCR. The stacked spheres at the bottom correspond to the catalyst substrate, where blue spheres represent nitrogen atoms, red spheres represent oxygen atoms, and white spheres represent hydrogen atoms. Black arrows illustrate the migration, adsorption, reaction and desorption pathways of reactants and products. In the left L-H mechanism, circled number ① describes the adsorption of gaseous NH_3_ on catalyst active sites, circled number ② refers to the adsorption of gaseous NO molecules, and circled number ③ denotes the surface reaction between pre-adsorbed NH_3_ and adsorbed NO to generate reaction intermediates. In the right E-R mechanism, circled number ① stands for the adsorption of NH_3_ on surface sites, while circled number ② indicates the reaction between adsorbed NH_3_ and gas-phase NO to yield N_2_ and H_2_O [63], (**b**) possible model of FC and WCO-FC catalysts PbO-resistant mechanism for NH_3_-SCR reaction [69], (**c**) proposed arsenic-resistant mechanism of the CeO_2_-WO_3_-Al_2_O_3_ catalyst [71], (**d**) the proposed possible K-resistant mechanism over CT(CeTiO*_x_*) and FZCT (FeZrCeTiO*_x_*) for NH_3_-SCR [77].

Notably, flue gas H_2_O significantly aggravates SO_2_ toxicity: as a reaction medium, it promotes SO_2_ dissolution and dissociation, accelerating metal sulfate and ammonium salt formation; it also enhances the interaction between sulfate products and the catalyst surface, intensifying deposition and synergistic poisoning-blocking effects. As shown in Figure 2a, under H_2_O and SO_2_ coexistence, a large number of ammonium salt sediments form on Nb-Sn_0.3_CeO_x_ catalyst surfaces, significantly reducing NO*_x_* adsorption/activation capacity, inhibiting NH_3_ adsorption and subsequent reactions, and leading to sharp attenuation of NH_3_-SCR activity [63].

### 3.2. Heavy Metal Poisoning Mechanism

#### 3.2.1. Lead (Pb) Poisoning

Pb, a common heavy metal pollutant in industrial flue gases, exerts toxicity through a progressive pathway of “active site occupation-redox cycle destruction-structure collapse”. It is mainly deposited on the catalyst surface as PbO, PbCl_2_, PbSO_4_, etc., preferentially occupying core active sites (e.g., Ce, Mn) and directly hindering the adsorption and activation processes of NO and NH_3_ [52]. Additionally, Pb destroys the Fe^2+^ + Ce^4+^↔Fe^3+^ + Ce^3+^ reversible redox cycle in FeCe catalysts, reducing surface-active oxygen content by over 30%, ultimately leading to catalyst structure collapse and specific surface area reduction. Comparative experiments show that unpoisoned Ce-based catalysts follow both Langmuir-Hinshelwood (L-H) and Eley-Rideal (E-R) mechanisms, while Pb poisoning causes Pb species to deposit on core active sites (e.g., Ce, W), inhibiting NH_3_ and NO_2_ adsorption/reduction and retaining only part of the L-H pathway (Figure 2b) [69]. Early studies on lead poisoning of ceria-based systems have also confirmed that lead species can interact strongly with the CeO_2_ support, altering the interfacial electronic state and the oxygen storage capacity (OSC), thereby affecting the integrity of the catalytic reaction pathways.

#### 3.2.2. Cadmium (Cd) and Arsenic (As) Poisoning

Both Cd and As cause significant deactivation of cerium-based SCR catalysts by interacting with surface-active components, damaging active site integrity and redox stability, but their poisoning pathways and effects are element-specific due to differences in atomic structure and chemical activity. Cd poisoning features the synergy of surface performance deterioration and slight pore blockage: Cd species selectively deposit on the surface (without penetrating the bulk phase), strongly interacting with active sites to reduce chemically adsorbed oxygen and acid sites (Lewis, Brønsted), impairing redox activity and NH_3_ adsorption-activation capacity; meanwhile, Cd accumulation slightly blocks pores, inhibiting reactant diffusion. Cd poisoning also transforms the reaction mechanism from dual L-H/E-R to single L-H, reducing reaction kinetics and worsening activity [70].

As poisoning differs essentially from Cd, its core is the strong interaction with cerium-based catalyst active components to form thermodynamically stable metal arsenates, which is the main cause of denitrification activity decline (confirmed by numerous experiments) [71]. For CeO_2_-WO_3_-Al_2_O_3_ catalysts, As interacts strongly with Ce and Al, causing massive loss of Lewis acid sites; the Ce-Al interface synergy alleviates poisoning by restoring Lewis acid sites and releasing oxygen vacancies, which reversely verifies the toxic nature of the strong interaction between As and active components, as shown in Figure 2c [71]. Li et al. further found that As-Ce interaction destroys CeO_2_ and WO_x_ synergistic catalysis, reducing Ce^4+^ content and redox performance, ultimately decreasing denitrification activity [72].

#### 3.2.3. Zinc (Zn) and Mercury (Hg) Poisoning

Zn and Hg exhibit unique poisoning mechanisms, related to their species characteristics and flue gas conditions. Zn poisoning mainly involves Zn species interacting with catalyst active components, damaging composite structure integrity and inhibiting NO adsorption/activation and NH_3_ catalytic conversion. The poisoning intensity of Zn species decreases in the order: ZnCl_2_ > ZnSO_4_ > ZnO [51], and ZnO poisoning further aggravates SO_2_-induced bulk sulfate formation, weakening catalyst sulfur resistance. Hg poisoning mainly occurs through the formation of HgO on the catalyst surface, which weakly adsorbs on active sites and causes reversible deactivation. Its toxicity is significantly lower than that of Pb and Cd due to its high volatility at typical SCR temperatures [73,74].

### 3.3. Alkali Metal/Alkaline Earth Metal Poisoning Mechanism

Alkali metals (K, Na) are a core bottleneck limiting the industrial application of cerium-based catalysts in high-alkali flue gases. The toxic mechanisms of these metals share commonalities with those of alkaline earth metals, but their toxicity is greater, and their effects are more comprehensive [75,76]. First, K, Na and other alkali metal species preferentially deposit on the catalyst surface, covering Ce-based core active sites and directly blocking NH_3_ adsorption and activation. As shown in Figure 2d, K poisoning significantly covers Ce active sites in the CeTiO*_x_* catalyst system, leading to a sharp decrease in NH_3_ adsorption capacity [77]. Second, alkali metals cause the disappearance of surface oxygen vacancies in cerium-based catalysts, significantly reducing the Ce^4+^/Ce^3+^ redox cycle rate; the poisoning effect of K^+^ is 2–3 times that of Ca^2+^ [78]. More importantly, they preferentially combine with the Ce-O-Ti active structure, blocking electron transfer between Ce^3+^ and Ti^4+^, further inhibiting oxygen vacancy formation in CeO_2_-TiO_2_ catalysts and ultimately causing a sharp decline in redox performance [77,79].

In contrast, alkaline earth metals (Ca, Mg) have a milder poisoning effect on cerium-based catalysts, with the core pathway focusing on surface acid site neutralization and mild redox deterioration, without irreversible damage to the bulk crystal structure—their impact on catalyst activity is significantly weaker than that of alkali metals. Taking typical basic oxide CaO as an example, it easily accumulates on the catalyst surface and undergoes irreversible neutralization reactions with Lewis and Brønsted acid sites, directly weakening NH*_3_* adsorption and activation capacity and disrupting the core SCR denitrification pathway. Additionally, CaO deposition slightly interferes with the redox cycle of active components, reducing Ce^3+^/Ce^4+^ and Fe^3+^/Fe^2+^ ratios and surface-active oxygen migration/activation efficiency, resulting in mild redox deterioration—this process does not cause complete active site deactivation or bulk structure damage [75,80,81].

### 3.4. Inhibitory Effect of H_2_O

Water vapor affects Ce-based catalysts via two completely different microscopic mechanisms, which are strictly dependent on water volume fraction.

When H_2_O content is lower than 8 vol%, it belongs to competitive adsorption inhibition: H_2_O molecules have a similar polarity to NH_3_, so they compete with NH_3_ for Lewis acid sites on the catalyst surface. This does not destroy the catalyst structure, but only reduces the effective adsorption quantity of reactants [82,83]. This competitive adsorption mechanism has been jointly verified by density functional theory (DFT) calculations and in situ characterizations conducted by European and American research groups. The research group led by international research teams performed DFT simulation calculations for the adsorption of NH_3_ probe molecules on W-doped CeO_2_ systems. The results confirmed that H_2_O occupies the acidic sites on the surface of Ce-based materials and drastically reduces the adsorption capacity of weakly adsorbed NH_3_. Compared with pure CeO_2_, the saturated adsorption capacity of NH_3_ on tungsten-modified ceria decreases by up to 21% after water vapor introduction. The intrinsic mechanism underlying site competition between water vapor and ammonia at low temperatures was elucidated from the perspective of electronic structure, and this conclusion is highly consistent with the experimental observations of the Ce-Cu-Ti composite oxide system.

Meanwhile, overseas research groups analyzed the co-adsorption behavior of multi-component gases on CeZr-based nanocluster models. Their DFT calculation results directly demonstrate that H_2_O anchors at Ce/Zr sites via its oxygen atoms, leading to direct site competition with NH_3_, which adsorbs through lone electron pairs on its nitrogen atoms. Moreover, this competitive interaction only alters the orientation of adsorbed molecules without inducing framework deformation of the oxide nanoclusters, solidifying the viewpoint that low-concentration water vapor merely triggers physical adsorption competition and causes no structural damage to the catalyst bulk phase.

**Figure 3:**
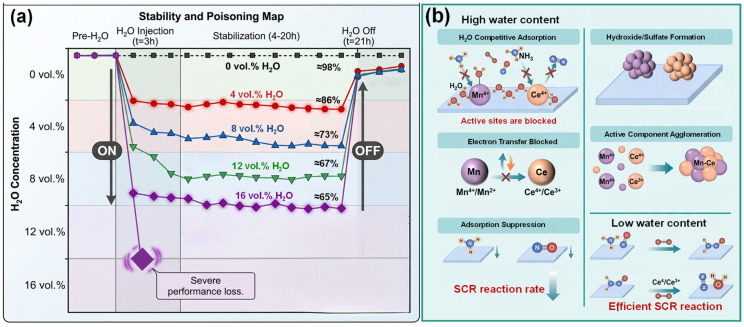
(**a**) time-dependent NO conversion curves at various H_2_O volume fractions, (**b**) schematic diagram for the deactivation mechanism of Mn-Ce catalysts under high H_2_O content and efficient SCR pathway under low H_2_O content. Light-blue slabs represent catalyst substrate; purple spheres are Mn sites and orange spheres are Ce sites. Blue-white clusters stand for NH_3_, red-white clusters for H_2_O, and blue-red molecules for NO. Red crosses mark blocked adsorption, electron transfer and reaction channels. Thin arrows illustrate molecular adsorption and electron migration, while thick downward arrows show reduced SCR reaction rate. Separate panels display H_2_O competitive adsorption, blocked electron transfer, suppressed reactant adsorption, hydroxide/sulfate formation and active component agglomeration at high water levels, alongside unimpeded catalytic cycles under low water conditions [84].

When H_2_O content exceeds 8 vol%, the mechanism transforms into chemical interference: a large number of H_2_O molecules adsorb on Mn^4+^ and Ce^4+^ active sites, block the electron transfer between Mn^4+^/Mn^2+^ and Ce^4+^/Ce^3+^, and inhibit the re-oxidation of low-valence metal ions (Figure 3b). As a result, the generation of key reaction intermediates (NH_3_(ad), NO(ad)) is reduced, and the overall SCR reaction rate declines sharply. In addition, high humidity accelerates the formation of sulfates and hydroxides and causes active component agglomeration. As H_2_O volume fraction increases from 0 vol% to 16 vol%, the NO conversion rate decreases concentration-dependently: ~98% under anhydrous conditions, ~86% at 4 vol% H_2_O, ~73% at 8 vol% H_2_O, ~67% and ~65% at 12 vol% and 16 vol% H_2_O [84,85].

### 3.5. Poisoning Mechanisms of Other Pollutants

P, HCl, HCHO and chlorobenzene also deactivate catalysts through specific mechanisms [86], with significant differences in their poisoning pathways, whose specific characteristics and mechanisms are as follows.

The poisoning effect of phosphorus species on Ce/TiO_2_ catalysts is essentially determined by the interaction mode between phosphorus and Ce active sites. Specifically, phosphorus reacts with Ce^4+^ to form thermodynamically stable CePO_4_, which directly blocks the NH_3_-SCR active sites and irreversibly disrupts the Ce^3+^/Ce^4+^ redox cycle, ultimately leading to a significant reduction in denitration activity. The introduction sequence of phosphorus serves as the key factor determining the intensity of the poisoning effect by regulating the contact timing and interaction strength between phosphorus and Ce active sites. International research teams have conducted quantitative tests on phosphorus poisoning of catalyst systems. The full experimental data and mechanistic conclusions can corroborate and expand the theoretical framework of phosphorus poisoning over cerium-based catalysts from the perspectives of toxicity classification of phosphorus species, multivariate regulation principles and differentiation of poisoning pathways, which greatly enhances the universality and engineering reference value of the relevant mechanistic discussion [87,88].

**Figure 4:**
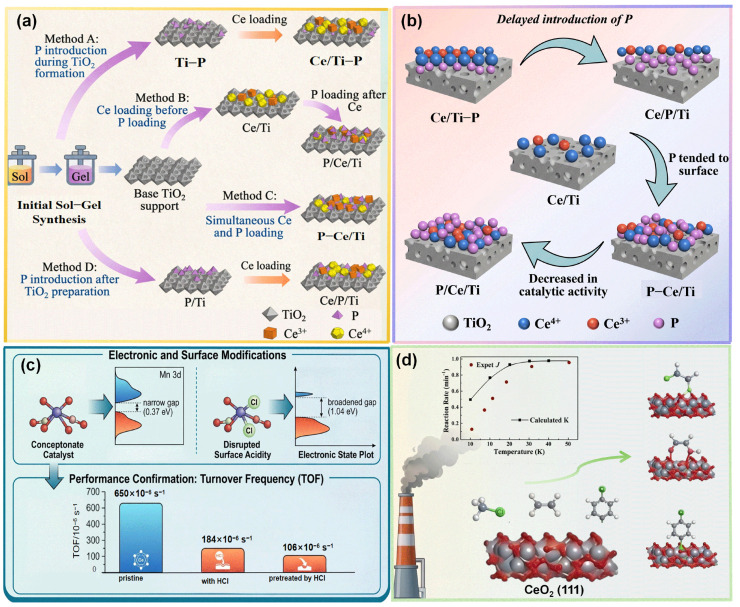
(**a**,**b**) schematic illustration of the influence of different phosphorus introduction sequences on the structure and catalytic performance of Ce/TiO_2_ catalysts [89], (**c**) schematic diagram of electronic and surface structural distortion over Mn-Ce catalysts under HCl poisoning. Blue and orange filled profiles represent Mn 3d electron orbitals; gradient blue arrows indicate electronic band evolution after Cl adsorption. The left region shows the initial narrow band gap of 0.37 eV, and the right region exhibits the broadened band gap of 1.04 eV induced by Cl doping, which disrupts surface acid sites and the intrinsic electronic structure of catalyst [95], (**d**) schematic illustration of the deactivation mechanism of ceria-based catalysts induced by toluene [94].

Figure 4a,b compares the structural evolution and poisoning behaviors of catalysts under four typical phosphorus introduction pathways. In the P/Ce/Ti pathway (phosphorus introduced after Ce loading), phosphorus directly reacts with the exposed Ce active sites to form CePO_4_, completely blocking the active sites and disrupting the redox cycle, resulting in a NO*_x_* conversion rate drop of over 30% [89,90], which represents the most severe poisoning effect. In the Ce/Ti-P pathway (phosphorus introduced during TiO_2_ preparation), phosphorus pre-modifies the support and is fixed in the support layer, avoiding direct contact with Ce active sites. Meanwhile, it optimizes the dispersion of Ce, effectively mitigating the negative effects of phosphorus. For the Ce/P/Ti pathway (phosphorus introduced after TiO_2_ preparation, followed by Ce loading), the pre-loaded phosphorus tends to migrate to the surface, which instead exacerbates the coverage of Ce active sites and intensifies poisoning. The P-Ce/Ti pathway (phosphorus introduced synchronously with CeO_2_ loading) exhibits a moderate poisoning effect, with no obvious alleviation. These results clearly reveal the core regulatory logic of phosphorus poisoning: only by fundamentally avoiding the direct contact between phosphorus and Ce active sites can the formation of CePO_4_ be blocked and phosphorus poisoning be avoided at the root. This provides clear theoretical guidance for the optimization of preparation processes for high phosphorus-resistant Ce-based SCR catalysts.

The poisoning mechanism of HCl on cerium-based catalysts involves two core dimensions: electronic structure regulation and catalytic activity attenuation (Figure 4c). For active component stability, HCl irreversibly reacts with surface CeO_2_ to form CeCl_3_, causing Ce loss and agglomeration and impairing intrinsic catalytic activity. For electronic structure and surface properties, HCl replaces bridge oxygen on Mn sites, transforming O-Mn-O into O-Mn-Cl, which significantly adjusts the electronic state distribution of Mn (3d orbital band gap increases from 0.37 eV to 1.04 eV) and destroys the electronic structure and surface acid sites of the catalyst. The turnover frequency (TOF) curve (Figure 4c) confirms this: the original catalyst’s TOF is ~650 × 10^−6^ s^−1^, which drops sharply after HCl treatment, leading to significant denitrification activity attenuation [91,92].

HCHO induces deactivation of Ce_x_Zr_1-x_O_y_ cerium-based catalysts through dual intrinsic and extrinsic pathways, closely related to catalyst chemical properties and structural evolution during interaction [93]. Extrinsically, HCHO oxidation on the catalyst surface generates by-products, such as C_6_H_9_CeO_6_ and Ce(CO_3_)_2_.

These substances deposit on the catalyst and cover active sites, blocking the contact between HCHO and active centers, which indirectly lowers catalytic activity. Intrinsically, HCHO triggers the reduction in Ce^4+^ to Ce^3+^, leading to a decline in Ce^4+^ content. As Ce^4+^ plays a critical role in oxygen migration and redox cycles, this change directly impairs the redox capability and ultimately results in catalyst deactivation.

Studies confirm that stable hydrocarbon intermediates formed from chlorobenzene on cerium-based catalysts can continuously adsorb and cover the catalytically active sites. Even under mild 580 K pyrolysis, their accumulation blocks reactant-active site contact, reducing catalytic activity. Additionally, defect sites (e.g., oxygen vacancies) promote initial C-Cl bond cleavage but preferentially adsorb these intermediates, further intensifying active site coverage and accelerating poisoning (Figure 4d) [94].

### 3.6. Multi-Pollutant Synergistic Poisoning Mechanism

Pollutants such as SO_2_, heavy metals (e.g., Pb, Cd, As), alkali metals/alkaline earth metals, and H_2_O present in industrial flue gases do not act on cerium-based catalysts independently in a single form, but form significant synergistic poisoning through physical-chemical coupling. Its toxicity intensity is much higher than the sum of single-pollutant toxicity, which is the core reason for the rapid deactivation and poor long-term stability of cerium-based catalysts in actual industrial conditions.

First, synergistic deterioration of acidic pollutants and polar molecules: Compared with single SO_2_ and H_2_O, their coexistence significantly accelerates ammonium salt sediment formation and deposition, greatly increasing catalyst pore blockage rate and hindering reactant diffusion, strengthening sulfate chemical poisoning on active sites, ultimately increasing denitrification activity attenuation rate several times. Meanwhile, H_2_O promotes adsorption and diffusion of heavy metal/alkali metal ions on the catalyst surface, intensifying active site occupation and structural damage, forming an “acidic pollutants + polar molecules” synergistic toxicity cycle. Studies confirm that SO_2_-H_2_O coexistence causes irreversible deactivation, much stronger than single pollutants, mainly due to their synergistic promotion of sulfate/ammonium salt deposition and weakening of catalyst redox capacity [96].

**Figure 5:**
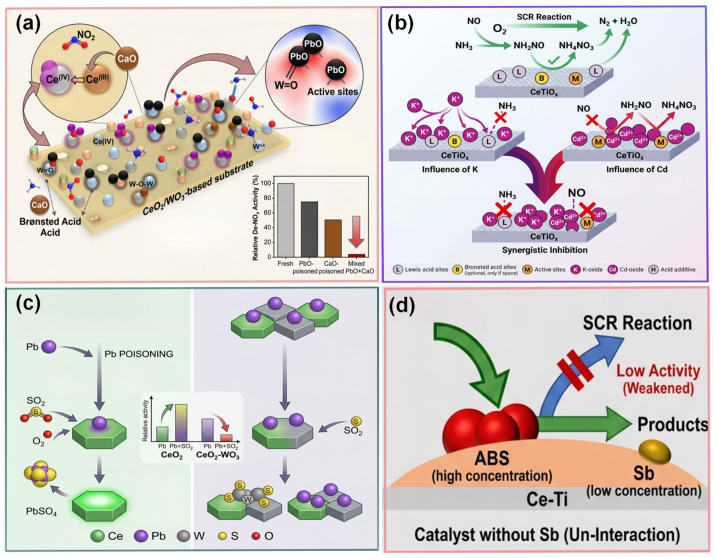
Schematic illustrations of multi-pollutant synergistic poisoning mechanisms on cerium-based NH_3_-SCR catalysts. (**a**) co-poisoning mechanism of CaO and PbO over CeO_2_/WO_3_-based catalyst, with insets showing the disturbed Ce redox cycle and PbO-covered active sites, and an embedded bar chart comparing relative De-NO*_x_* activity under different poisoning conditions [97], (**b**) synergistic inhibition effect of K and Cd co-poisoning on CeTiO*_x_* catalyst, including the SCR reaction pathway, individual poisoning effects of K and Cd, and the synergistic inhibition state [98], (**c**) interaction mechanism of Pb and SO_2_ over CeO_2_ and CeO_2_–WO_3_ catalysts, with an embedded bar chart comparing relative activity under Pb-only poisoning and Pb–SO_2_ co-poisoning [46], (**d**) synergistic poisoning effect of Sb and ammonium bisulfate (ABS) on Ce–Ti catalyst. Legend and symbol description: L = Lewis acid sites, B = Brønsted acid sites, M = active sites; K-oxide = potassium oxide species, Cd-oxide = cadmium oxide species; red cross = inhibition of corresponding sites or reaction steps; purple arrow = combined action of multiple poisons; downward red arrow = decrease in catalytic activity; green arrow = reaction pathway; red double-barred arrow = weakened reaction. Element markers: Ce (green), Pb (purple), W (gray), S (yellow), O (red) [99].

Second, the superimposed poisoning of metal pollutants: Coexisting alkali/alkaline earth metals and heavy metals (Pb, Cd, As) form a multi-metal composite poisoning layer on the catalyst surface: alkali metals neutralize surface acid sites and inhibit NH_3_ adsorption; heavy metals destroy the Ce^3+^/Ce^4+^ redox cycle and cause crystal structure collapse. Their synergy leads to irreversible dual declines in active site quantity and redox performance. For example, Figure 5a schematically illustrates the CaO–PbO co-poisoning mechanism on CeO_2_/WO_3_-based catalysts: the two enlarged insets respectively depict the CaO-disturbed Ce(IV)/Ce(III) redox cycle and PbO-blocked active sites, accompanied by weakened W=O sites and reduced Brønsted acid sites on the substrate surface; the embedded bar chart compares relative De-NO*_x_* activity under fresh, single Pb-poisoned, single CaO-poisoned and mixed PbO–CaO poisoned conditions, with the downward red arrow marking the synergistic activity decline. Ca promotes Ce^3+^ to Ce^4+^ conversion to inhibit redox cycles, while Pb blocks active sites and intensifies surface element electronegativity changes, synergistically reducing activity [97]. Similarly, Figure 5b demonstrates the K–Cd synergistic inhibition on CeTiO*_x_* catalysts: the upper green arrows describe the standard NH_3_-SCR reaction pathway via NH_2_NO and NH_4_NO_3_ intermediates; red cross symbols indicate the blocking effect of K-oxide on Lewis acid sites, and the suppression of Cd-oxide on active sites and nitrate intermediate formation; the purple arrow represents the combined action of two poisons that simultaneously damage acid sites and reactive sites. Consequently, K–Cd coexistence significantly inhibits NH_3_ adsorption and active nitrate intermediate generation, reducing denitrification efficiency. K destroys Lewis acid sites, and Cd inhibits intermediate generation, synergistically aggravating deactivation [98].

Third, a complex interaction between acidic and metal pollutants: Some alkali metals can react with SO_2_ to form sulfate species, which alleviate the neutralization effect of alkali metals on catalyst acid sites to a certain extent, showing a slight antagonistic effect; but the generated sulfates will further block the catalyst pores and strengthen physical poisoning, and the overall effect is dominated by synergistic toxicity. The coexistence of SO_2_ and heavy metals will form stable metal sulfate precipitates, which not only directly occupy the catalyst active sites but also destroy the catalyst crystal structure, forming irreversible dual chemical-physical synergistic poisoning and greatly shortening the service life of the catalyst. As shown in Figure 5c, the interaction between SO_2_ and Pb on cerium-based catalysts exhibits distinct behaviors on different supports: purple spheres denote Pb species, green hexagons denote Ce active sites, gray spheres denote W sites, and yellow/red spheres denote S and O elements, respectively; black arrows indicate the evolution path from Pb poisoning to PbSO_4_ formation in the presence of SO_2_ and O_2_; the embedded bar chart quantitatively compares relative activity under single Pb poisoning and Pb + SO_2_ co-poisoning for both CeO_2_ and CeO_2_−WO_3_ catalysts, with the red downward arrow indicating the activity decline trend. For the catalyst with only CeO_2_ active sites, the reaction of Pb with SO_2_ forms PbSO_4_, which can restore the activity of partially poisoned CeO_2_ active sites. In contrast, on the CeO_2_–WO_3_ catalyst with dual-active sites, SO_2_ poisons the acidic sites, and Pb deactivates the active sites; their combined action aggravates catalyst poisoning. This further demonstrates the complexity of the interaction between the two substances [46]. In addition, the synergistic poisoning of Sb and ammonium bisulfate (ABS) on the surface of cerium-based catalysts presents similar characteristics, as illustrated in Figure 5d. The thick green arrow represents the normal SCR reaction pathway over the Ce–Ti catalyst; the red double-barred arrow indicates that the SCR reaction is significantly weakened (low activity) under high-concentration ABS coverage and low-concentration Sb modification. The strong interaction between the two will significantly damage the catalyst structure and redox capacity, while enhancing the thermal stability of ABS, intensifying pore blockage and active site coverage [99].

In summary, multi-pollutant poisoning on cerium-based catalysts essentially involves the superposition of chemical and physical effects. Chemical effects mainly include specific reactions between pollutants and catalyst active components, destroying active site integrity and interrupting the Ce^3+^/Ce^4+^ redox cycle; physical effects focus on pollutant deposition on the catalyst surface, causing pore blockage and active site coverage. The two synergistically accelerate cerium-based catalyst deactivation.

The deactivation degree and intrinsic changes of cerium-based catalysts vary greatly under different flue gas poisoning conditions. The key characteristic parameters of catalysts under typical single and combined poisoning conditions are listed in Table 1.

## 4. Modification Strategies and Performance Optimization of Cerium-Based Catalysts

Cerium-based catalysts have inherent defects and are susceptible to deactivation due to poisoning by various impurities found in industrial flue gas. In response, the academic community has developed various targeted modification strategies, the most central and technically mature of which is elemental doping. Introducing rare earth, transition metal, and non-metallic elements optimizes the catalytic performance in terms of electronic structure regulation, surface physicochemical property tuning, and crystal structure reconstruction. These modifications effectively enhance the catalysts’ catalytic activity, stability, and anti-poisoning capability, providing a critical foundation for their industrial application.

### 4.1. Rare Earth Element Doping Modification

Rare earth elements, with atomic radii and electronic configurations similar to Ce, generate strong synergistic effects when doped. By regulating electronic structure, optimizing surface acid sites, and inducing oxygen vacancies, the physicochemical properties of cerium-based catalysts are improved, significantly enhancing NH_3_-SCR denitrification performance and anti-poisoning capacity. Currently, La, Pr, Nd, Sm, and Y are the most widely used rare earth dopants, with distinct regulatory effects due to different electronic structures [100,101,102].

As a representative dopant, La can remarkably enhance the structural stability and anti-poisoning performance of cerium-based SCR catalysts. Existing as stable La^3+^, its good chemical stability suppresses Ce-based active component agglomeration, promotes surface oxygen vacancy generation and enrichment, and provides sufficient active sites.

**Figure 6:**
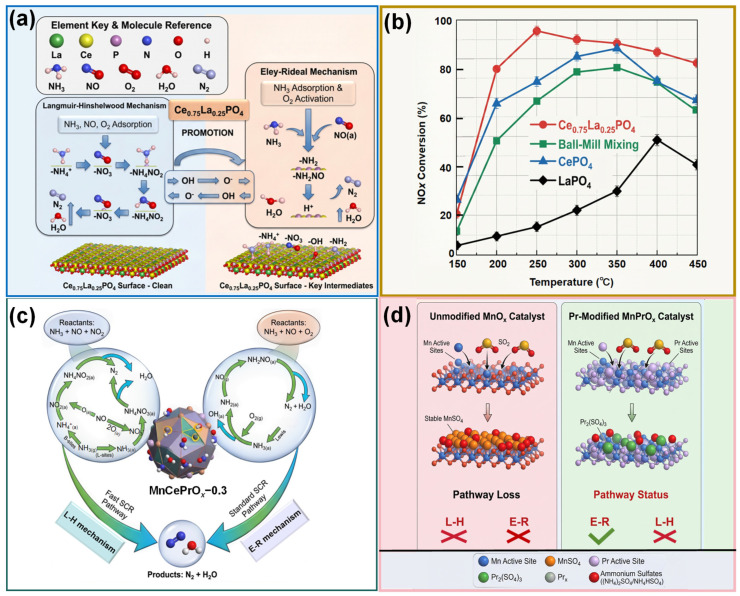
(**a**) schematic diagram of NH_3_-SCR reaction mechanism over Ce_0.75_La_0.25_PO_4_ catalyst (L-H and E-R synergistic mechanism) [103], (**b**) NO*_x_* conversion curves of different Ce/La-based catalysts as a function of reaction temperature [103], (**c**) schematic diagram of dual reaction pathways for NH_3_-SCR over MnCePrO*_x_* catalyst. Green curved arrows denote elementary reaction steps, blue arrows stand for the overall reaction direction toward final products N_2_ and H_2_O, (**d**) schematic of the promoting effect and reaction mechanism of Pr modification on SO_2_ resistance over MnO*_x_* catalyst [106], green tick denotes intact E-R pathway, red cross represents blocked L-H or E-R pathways.

Tan et al. synthesized Ce_0.75_La_0.25_PO_4_ via La doping and clarified its NH_3_-SCR mechanism (Figure 6a), which follows both L-H and E-R pathways, adsorbs NO and NH_3_ from intermediates (-NH_4_NO_2_, -NH_2_NO) before converting to N_2_ and H_2_O; La facilitates oxygen vacancy formation and active oxygen migration to maintain the redox cycle [103]. Catalytic tests (Figure 6b) showed Ce_0.75_La_0.25_PO_4_ maintained ≥95% NO*_x_* conversion at 150–300 °C, outperforming pure CePO_4_, LaPO_4_, and their mixtures. Characterization revealed a 37% higher specific surface area and 2.1-fold higher oxygen vacancy concentration than pure CePO_4_. This finding is consistent with the work of Li et al., who used La-Mn-Fe composite-modified activated carbon and achieved NO*_x_* conversion exceeding 90% in a similar temperature window [103,104].

Unlike La, Pr features the Pr^3+^/Pr^4+^ redox couple, enabling stronger electronic synergy with Ce, higher oxygen vacancy density, and better active component dispersion, leading to superior modification effects. Wang et al. prepared Pr-modified MnCeO*_x_* catalysts and systematically clarified the dual-path reaction mechanism and sulfur resistance enhancement mechanism. Figure 6c presents the NH_3_-SCR dual reaction pathways over MnCePrO*_x_* catalyst: the reaction proceeds via both Langmuir-Hinshelwood (L-H) and Eley-Rideal (E-R) mechanisms. Curved green arrows inside the circular modules represent elementary reaction steps of each pathway; blue arrows connecting the modules to the product zone indicate the overall conversion direction toward N_2_ and H_2_O products. In the L-H pathway, gas-phase NH_3_ is anchored on Brønsted (B-sites) and Lewis acid sites (L-sites) to form adsorbed NH_3_ species, which rapidly react with adsorbed NO_2_ intermediates following the fast SCR route. In the E-R pathway, NH_3_ adsorbed on Lewis acid sites reacts with gas-phase NO through the standard SCR route. The synergistic operation of the two pathways significantly accelerates the overall reaction rate [42,105]. Figure 6d interprets the intrinsic SO_2_ tolerance mechanism. For pristine Mn-based catalysts, SO_2_ reacts directly with Mn active sites to form stable MnSO_4_, accompanied by the deposition of ammonium sulfate species on the surface, which severely blocks both L-H and E-R reaction channels (marked by red crosses in the schematic) and leads to irreversible deactivation. After Pr modification, PrO_x_ species preferentially react with SO_2_ to generate Pr_2_(SO_4_)_3_, acting as a sacrificial phase to protect Mn active centers from sulfation. Consequently, the E-R reaction pathway remains effectively functional (marked by a green check mark), and the inhibition of the L-H pathway is also notably weakened (marked by a red cross) [106]. Benefiting from the above effects, the catalyst maintained NO conversion above 90% at 250 °C and achieved over 90% NO*_x_* conversion across 90–270 °C in 100 ppm SO_2_-containing simulated flue gas, exhibiting excellent low-temperature activity and sulfur-poisoning resistance.

Besides La and Pr, Nd, Y, and Sm also show excellent modification effects on cerium-based catalysts, with distinct mechanisms and performance advantages: Nd doping forms a stable solid-solution structure with Ce. Nd atoms enter the CeO_2_ lattice, inducing lattice expansion, promoting Ce^4+^ reduction to Ce^3+^ and oxygen vacancy migration. It also converts Lewis acid sites to Brønsted acid sites, strengthening NH_3_ adsorption/activation and NO oxidation to NO_2_ (providing fast SCR intermediates). Experiments show Nd-doped catalysts achieve >90% NO*_x_* conversion at 200 °C, with improved low-temperature activity and reaction rate [107]; additionally, they accelerate redox cycles, optimize surface charge balance, and provide more active sites.

**Figure 7:**
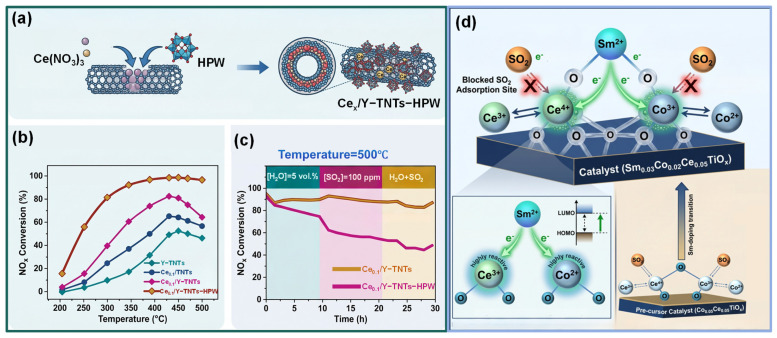
(**a**–**c**) preparation process, denitration activity, and H_2_O/SO_2_ resistance performance of Y-doped Ce-based titania nanotube composite catalysts [21], (**d**) schematic diagram of the regulation mechanism of Sm doping on the sulfur poisoning resistance of Ce-Co-TiO_x_ catalysts [109]. Green arrows denote electron transfer between metal sites; upward green arrow in the band structure diagram represents promoted electron excitation from HOMO to LUMO; red cross marks indicate blocked SO_2_ adsorption.

Y doping causes CeO_2_ lattice distortion via ionic radius mismatch, realizing dual regulation: it modulates redox behavior and acid site distribution and improves hydrothermal stability for industrial flue gas conditions. As shown in Figure 7a,b, the Ce_x_/Y-TNTs-HPW composite catalyst (prepared by loading Ce and HPW on Y-TNTs) achieves nearly 100% NO*_x_* conversion at 200–500 °C, far outperforming unmodified counterparts [21,108]. It retains excellent performance under harsh conditions (500 °C, 5 vol.% H_2_O and 100 ppm SO_2_), attributed to increased chemisorbed oxygen, enhanced redox cycles, and optimized acidity/oxygen vacancies (Figure 7c).

Sm doping synergistically improves catalytic activity and sulfur resistance via Sm^2+^/Sm^3+^ and Ce^4+^/Ce^3+^ electron transfer cycles. Green arrows represent electron transfer between metal sites, and the upward green arrow in the HOMO-LUMO diagram stands for narrowed band gap and facilitated electron excitation. These cycles strengthen NO*_x_* adsorption and NH_3_ activation while promoting the formation of -NH_2_, and they inhibit SO_2_-induced electron transfer to reduce sulfate poisoning. The Sm_0.03_Co_0.02_Ce_0.05_TiO_x_ catalyst avoids deep sulfuration in contrast to Sm-free counterparts (Figure 7d) [109]. Sm-doped CeO_2_-TiO_2_ maintains over 80% NO conversion after 32 h of operation at 250 °C under the atmosphere containing 5 vol.% H_2_O and 200 ppm SO_2_, with an active temperature window of 180–460 °C [110]. Sm also enhances oxygen storage capacity; in the Ce-Sm/Cu-SSZ-13 catalyst, it promotes the conversion of NH_4_NO_3_ to NH_4_NO_2_, strengthening low-temperature denitrification efficiency with NO*_x_* conversion reaching 93.1% at 200 °C [111].

### 4.2. Modification of Cerium-Based Catalysts by Transition Metal Elements

Although doping with rare earth elements can effectively improve the denitrification performance and anti-poisoning ability of cerium-based catalysts, there are still obvious inherent drawbacks. First, the low-temperature activity (100–200 °C) of most rare earth-doped catalysts cannot currently meet the denitrification requirements of low-temperature industrial flue gas. Second, the effect of doping with a single rare earth element on enhancing the surface redox cycle of catalysts is limited, making them prone to activity attenuation in complex flue gas. Third, after doping with certain rare earth elements, the catalysts have a narrow temperature active window, making them difficult to adapt to the fluctuating temperature conditions of industrial flue gas. Therefore, a key route to further optimizing the comprehensive performance of cerium-based catalysts and promoting their industrial application is to modify them by introducing typical transition metal elements (such as Mn, Fe and Cu) and utilizing the synergistic effect between transition metals, Ce and rare earth elements to target the above defects.

#### 4.2.1. Mn Doping

**Figure 8:**
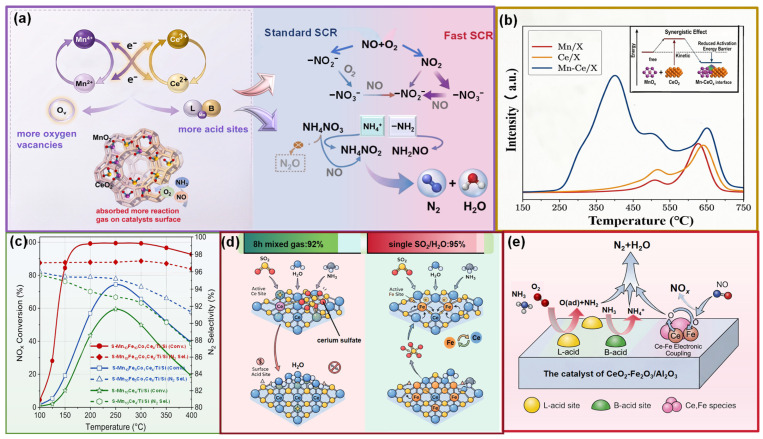
(**a**) schematic diagram of the multi-level redox cycle and SCR reaction mechanism over Mn-Ce based catalyst [112], (**b**) H_2_-TPR profiles of X zeolite-supported Mn/X, Ce/X and Mn-Ce/X catalysts, with the inset schematic illustrating the synergistic electronic interaction at the Mn-CeO*_x_* heterointerface that reduces the reaction activation energy barrier [112], (**c**) NO*_x_* conversion and N_2_ selectivity profiles of S-MnCoCe/Ti/Si catalysts with different Fe doping amounts as a function of temperature [114], (**d**) schematic comparison of sulfur-water poisoning mechanisms over pristine Ce-based and Fe-doped Ce-based catalysts [114], arrows represent adsorption, migration and transformation of gas species; prohibition symbols indicate blocked surface acid sites. (**e**) schematic diagram of the synergistic denitration mechanism between surface acid sites and Ce-Fe active species over CeO_2_-Fe_2_O_3_/Al_2_O_3_ catalyst [115]. Arrows denote the evolution of reactants and intermediates toward final products N_2_ and H_2_O.

Manganese (Mn) and cerium (Ce) are widely recognized as the “golden combination” in the field of low-temperature SCR denitrification. Their synergistic effect can specifically address the prevalent shortcoming of insufficient low-temperature activity for single rare-earth-doped cerium-based catalysts. The core mechanism lies in the construction of a dynamic electron transfer system centered on the equilibrium Mn^3+^ + Ce^4+^↔Mn^4+^ + Ce^3+^, which establishes multi-stage redox cycles of Mn^4+^/Mn^2+^ and Ce^3+^/Ce^4+^ simultaneously. This unique dual-active-site structure not only significantly increases the oxygen vacancy concentration on the catalyst surface but also further optimizes acid site density via manganese introduction. Collectively, these effects promote the adsorption and activation of ammonia and nitric oxide (NO), effectively compensate for the sluggish reaction kinetics of single rare earth doping at low temperatures, and drive both the standard SCR and fast SCR pathways efficiently (Figure 8a) [112].

The intrinsic redox enhancement arising from Mn-Ce synergy can be directly reflected by H_2_-TPR characterization, as shown in Figure 8b [112]. For the pristine zeolite X support and single metal-loaded samples (Mn/X, Ce/X), their reduction peaks mainly appear in the medium-high temperature region, with weak low-temperature reducibility. In comparison, the Mn-Ce co-loaded sample exhibits a remarkable shift in reduction peaks toward lower temperatures and an obvious increase in total hydrogen consumption. This result indicates that strong interfacial electronic interaction occurs between MnO*_x_* clusters and cerium species on the support surface, which lowers the reduction energy barrier of active metal species and accelerates the redox cycle process, providing direct experimental evidence for the electron transfer mechanism illustrated in Figure 8a.

Support type and preparation method are core variables that further regulate the performance of Mn-Ce-rare-earth-based catalysts, directly determining the final catalytic activity and operational stability. In terms of preparation method regulation, this factor not only governs the microstructure of the catalyst but also serves as a key strategy to alleviate the insufficient low-temperature stability of rare-earth-doped systems. Yao et al. systematically compared five preparation routes: mechanical mixing, impregnation, hydrothermal synthesis, coprecipitation, and the sol-gel method. The results demonstrate that hydrothermal synthesis is most favorable for incorporating Mn^*n*+^ (*n* = 2–4) into the ceria lattice to form a homogeneous Ce-Mn-O solid solution, raising the Ce^3+^ proportion to 18.67% and thus significantly improving oxygen vacancy concentration and redox cycle efficiency. Benefiting from this structural advantage, the catalyst achieves nearly 100% NO conversion over a wide-temperature window of 100–325 °C. Even after 45 h of continuous operation at 200 °C in an atmosphere containing 5 vol.% H_2_O, its NO conversion remains at 80–90%, effectively overcoming the common drawbacks of facile low-temperature deactivation and poor water resistance for single rare-earth-doped catalysts [113].

#### 4.2.2. Fe Doping

The core advantage of Fe doping modification is to targetedly remedy the inherent defects of single rare earth-doped cerium-based catalysts, such as a narrow temperature active window and limited sulfur-water resistance. It endows Ce-based catalysts with excellent wide-temperature activity and strong sulfur-water resistance, while effectively making up for the weak lattice oxygen migration ability and insufficient surface acidity of pure Ce-based catalysts. Its main mechanism relies on electron transfer from Fe^2+^ to Mn^4+^, which inhibits the oxidation of SO_2_ by Mn^4+^ and reduces the deposition of toxic products on the catalyst surface. At the same time, it increases the total acidity of the catalyst and enhances the adsorption and activation of NH_3_, thereby improving the stability of the catalyst in complex flue gas environments and solving the problem of easy deactivation of single rare earth doping under high-sulfur and high-humidity conditions.

Qiu et al. optimized the S-MnCoCe/Ti/Si system via iron doping and systematically investigated the synergistic regulation effect of Fe doping amount on catalytic performance and reaction selectivity. The results revealed that the optimized S-Mn_10_Fe_10_Co_1_Ce_4_/Ti/Si catalyst achieved nearly 100% NO*_x_* conversion over a wide-temperature window of 150–300 °C, which was remarkably superior to samples with low Fe doping and single rare earth-doped counterparts. Meanwhile, the N_2_ selectivity remained steadily above 96% across the entire tested temperature range, an increase of approximately four percentage points compared with the unmodified catalyst. This modification effectively suppressed excessive oxidation of NH_3_ and the generation of by-products at high temperatures, enabling simultaneous improvement in catalytic activity and selectivity (Figure 8c) [114]. In terms of poisoning resistance, iron doping can reshape the sulfur and water tolerance mechanism of the catalyst at the microscopic level, and fundamentally alleviate the inherent drawback of single rare earth cerium-based catalysts that are prone to irreversible sulfur poisoning. Quantitative performance tests show that in an atmosphere coexisting with 50 ppm SO_2_ and 10 vol.% H_2_O, the optimized iron-modified catalyst can achieve an activity retention rate of 92% after 8 h of continuous reaction; the activity attenuation caused by SO_2_ alone or H_2_O alone is less than 5%, and the activity recovery rate exceeds 95% after removing the poisons, indicating that the overall poisoning process is dominated by reversible adsorption. The core mechanism of this performance improvement can be intuitively illustrated in Figure 8d. Arrows represent the adsorption, migration and transformation of gas molecules and surface species; circular prohibition symbols denote blocked surface acid sites. For the unmodified single cerium-based catalyst, SO_2_ tends to directly bind with Ce active sites to form stable cerium sulfate, while water molecules competitively adsorb on surface acidic sites, jointly leading to irreversible activity attenuation. After the introduction of iron species, Fe sites can preferentially capture SO_2_, reducing its erosion on Ce active centers. Meanwhile, the electron transfer between Fe and Ce weakens the stability of sulfate species, facilitating their decomposition and desorption. Coupled with the inhibitory effect of increased total surface acidity on the competitive adsorption of water molecules, the poisoning reversibility and operational stability are simultaneously improved [114].

To further reveal the enhancement mechanism of Fe doping on catalytic performance, researchers conducted an analysis using the CeO_2_-Fe_2_O_3_/Al_2_O_3_ model system. In this system, the Ce-Fe bimetallic active components and surface acidic sites form an efficient synergy. Brønsted acid sites and Lewis acid sites are responsible for the adsorption and activation of NH_3_ to generate NH_4_^+^ and -NH_2_ active species, respectively; curved arrows describe the conversion of reaction intermediates, and upward arrows stand for the formation of final products N_2_ and H_2_O. The electronic coupling between Ce and Fe accelerates NO oxidation and oxygen vacancy cycling, providing continuous redox power for the SCR reaction (Figure 8e) [115]. This microscopic mechanism remedies the inherent shortcoming of insufficient synergy of active sites in single rare earth-doped catalysts from the perspective of surface acidity-redox matching, making the catalytic reaction pathway more efficient and stable [95].

Based on this mechanism, researchers further optimized the performance of the Fe-Ce system through modification strategies to targetedly solve the problems of a narrow temperature active window and poor stability in complex flue gas of single rare earth-doped catalysts. Wang et al. modified the Fe-Ce catalyst with sulfate (obtaining the SFC sample). The regulation of sulfate significantly broadened the catalyst active window, extending its effective temperature range to 210–450 °C with NO*_x_* conversion above 90%, successfully remedying the insufficient wide-temperature performance of single rare-earth-doped catalysts. The Ce^3+^ ratio in this modified catalyst reaches 33.7%, and the surface adsorbed oxygen content is as high as 73.3%. This structural advantage ensures no obvious decrease in NO conversion in complex flue gas containing H_2_O and SO_2_ at 270 °C, making it suitable for denitrification scenarios of complex flue gas such as coal-fired boiler exhaust [116].

#### 4.2.3. Cu Doping

Copper doping modification can systematically remedy the inherent drawbacks of single rare-earth-doped ceria-based catalysts, including insufficiently reinforced electron transfer cycles, severe agglomeration of active components, weak poisoning resistance and limited functionality. A synergistic redox cycle of Cu^2+^ + Ce^3+^↔Cu^+^ + Ce^4+^ is constructed upon Cu doping, which greatly elevates the surface oxygen vacancy concentration and optimizes the electronic structure of active sites. Meanwhile, the strong interaction between Cu and Ce facilitates the homogeneous dispersion of active components, enlarges the specific surface area and raises the content of surface adsorbed oxygen (Oα). As a result, the catalyst gains superior resistance against erosion by sulfur dioxide and alkali metals, longer service life, as well as broader application scope and better adaptability.

**Figure 9:**
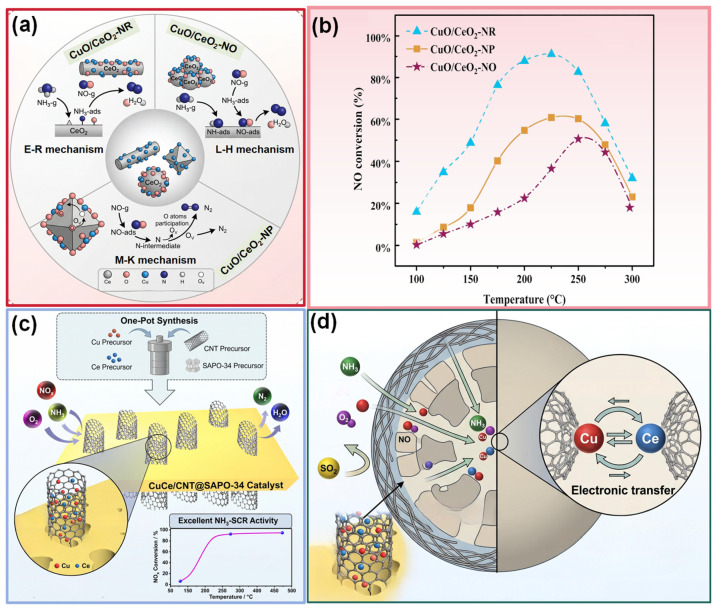
(**a**) schematic diagram illustrating different NH_3_-SCR reaction mechanisms over CuO/CeO_2_ catalysts with various morphologies (nanorod: NR, nanoparticle: NP, NO-preoxidized: NO); arrows represent adsorption, migration and conversion of gaseous reactants and intermediates [29], (**b**) schematic diagram of the microstructure and oxygen vacancy distribution of CuO/CeO_2_-NR, CuO/CeO_2_-NP and CuO/CeO_2_-NO catalysts [29], (**c**) schematic illustration of the hierarchical pore structure and electronic interaction in CuCe/CNT@SAPO-34 composite catalyst, arrows denote gas diffusion and reaction progress [118], (**d**) schematic diagram illustrating the anti-poisoning mechanism of CuCe/CNT@SAPO-34 composite catalyst under flue gas containing SO_2_ and H_2_O. The left half displays the selective diffusion behavior of gaseous reactants (NH_3_, NO, O_2_) and toxic SO_2_ molecules inside the hierarchical pore structure; the magnified inset on the right reveals the reversible electronic transfer cycle between Cu and Ce active species, arrows indicate gas diffusion direction and interfacial electron transfer between Cu and Ce [118].

To reveal the detailed promotion mechanism of Cu doping on low-temperature denitrification performance, Zhang et al. fabricated a series of CuO/CeO_2_ catalysts with diverse morphologies via the impregnation method and optimized their catalytic performance by morphology regulation. Benefiting from abundant oxygen vacancies and ordered microstructure, the nanorod-shaped CuO/CeO_2_-NR sample enables preferential adsorption of NH_3_ at Lewis acid sites anchored on Cu sites. Its NH_3_-SCR reaction proceeds following a combined mechanism dominated by the E-R pathway and assisted by the L-H pathway, arrows in Figure 9a depict the adsorption and transformation pathways of gaseous reactants and surface intermediates [29]. Catalytic activity tests demonstrate that the nanorod catalyst delivers much higher NO conversion than nanoparticulate (CuO/CeO_2_-NP) and NO-preoxidized (CuO/CeO_2_-NO) samples within 100–300 °C, achieving the maximum denitrification efficiency at 200 °C (Figure 9b) [29,117].

However, morphology regulation only solves low-temperature activity and dispersion problems, not the narrow wide-temperature active window and limited water-sulfur resistance of rare-earth-doped catalysts. To address this, researchers adopted a support composite strategy to prepare the CuCe/Carbon Nanotube (CNT)@Silicoaluminophosphate-34 (SAPO-34) composite catalyst via one-pot hydrothermal synthesis. The hierarchical pore structure of the CNT@SAPO-34 composite support alleviates active component agglomeration and low mass transfer efficiency, enabling excellent NH_3_-SCR activity in 200–450 °C, arrows in Figure 9c represent gas molecule diffusion and SCR reaction progress [118].

The intrinsic mechanism behind the improved poisoning resistance is schematically illustrated in Figure 9d. As revealed by relevant studies on CNT-modified molecular sieve catalysts [118], the performance enhancement stems from the synergistic effect of structural shielding and electronic modulation. Structurally, the interwoven CNT network and SAPO-34 microporous framework form a selective diffusion barrier: small reactant molecules such as NH_3_ and NO can readily pass through pore channels to access internal active sites, while the inward penetration of SO_2_ is effectively retarded, thus reducing the deposition of ammonium sulfate salts and the direct sulfation of metal active centers. Electronically, the electron-rich CNT promotes interfacial charge transfer between Cu and Ce species, elevates the proportion of high-valence active sites and surface chemisorbed oxygen, and mitigates irreversible deactivation caused by stable metal sulfate formation. In Figure 9d, straight arrows indicate gas diffusion direction, and circular bidirectional arrows stand for reversible electron transfer between Cu and Ce. Benefiting from this dual-effect enhancement, the catalyst maintains stable denitration efficiency under complex flue gas conditions and is well-suited for medium-low temperature industrial denitrification scenarios.

### 4.3. Non-Metal Element Doping Modification of Cerium-Based Catalysts

Although rare earth and transition metal doping have significantly improved the low-temperature activity and redox cycling performance of cerium-based catalysts, the catalysts still suffer from severe issues such as sulfate poisoning, remarkable water inhibition, easy grain sintering and active site coverage under the extreme operating conditions of sintering flue gas featuring high sulfur, high humidity, high temperature and coexistence of multiple poisons. Relying solely on metal regulation cannot fundamentally break through these bottlenecks at the surface and interface level.

Accordingly, doping with non-metal elements, including sulfur, nitrogen and phosphorus, has become an important extended strategy for cerium-based catalyst modification. This modification precisely regulates surface acidity, electronic structure, defect density and crystal phase stability, transforming traditional “poison components” into “active sites”. While maintaining high denitrification efficiency, it significantly enhances the anti-poisoning, water-resistant and anti-sintering capabilities of the catalysts in complex industrial flue gas, forming a complete modification system integrating metal doping and non-metal doping. On the basis of the above three mainstream non-metal dopants, boron and fluorine, two typical anionic non-metal elements, have also been widely studied in recent years, and their modification mechanisms and anti-poisoning characteristics are further supplemented below.

#### 4.3.1. Sulfur Doping Modification

In high-sulfur flue gas, SO_2_ easily induces the formation of inert sulfates on cerium-based catalysts and causes deactivation, which has long been a critical problem restricting their industrial application. The core breakthrough of sulfur doping lies in converting “sulfur poisons” into “catalytic active mediators”, blocking irreversible poisoning mechanistically, and simultaneously strengthening surface acidity and oxygen vacancy concentration, truly realizing “stable operation in high-sulfur atmospheres”. At present, two controllable pathways have been formed: gas-phase sulfidation and sulfate precursor doping. Gas-phase sulfidation constructs stable sulfate species on the catalyst surface via mild treatment in a SO_2_-O_2_ atmosphere, and promotes the conversion of Ce^4+^ to Ce^3+^, greatly increasing the concentration of oxygen vacancies and Brønsted acid sites. Taking the Ce_0.6_Zr_0.4_O_2_ system as an example, sulfidation not only significantly elevates the proportion of Ce^3+^ but also stabilizes performance through a dual mechanism: on the one hand, it inhibits the coverage of active sites by inert nitrates; on the other hand, it enhances the high-temperature adsorption stability of NH_3_, providing continuous active intermediates for the E-R reaction. The modified catalyst maintains a NO conversion rate of over 90% at 250–300 °C, and retains an efficiency of more than 85% after long-term operation in high-concentration SO_2_, fundamentally remedying the sulfur-susceptible deactivation defect of pure cerium-based catalysts [119].

**Figure 10:**
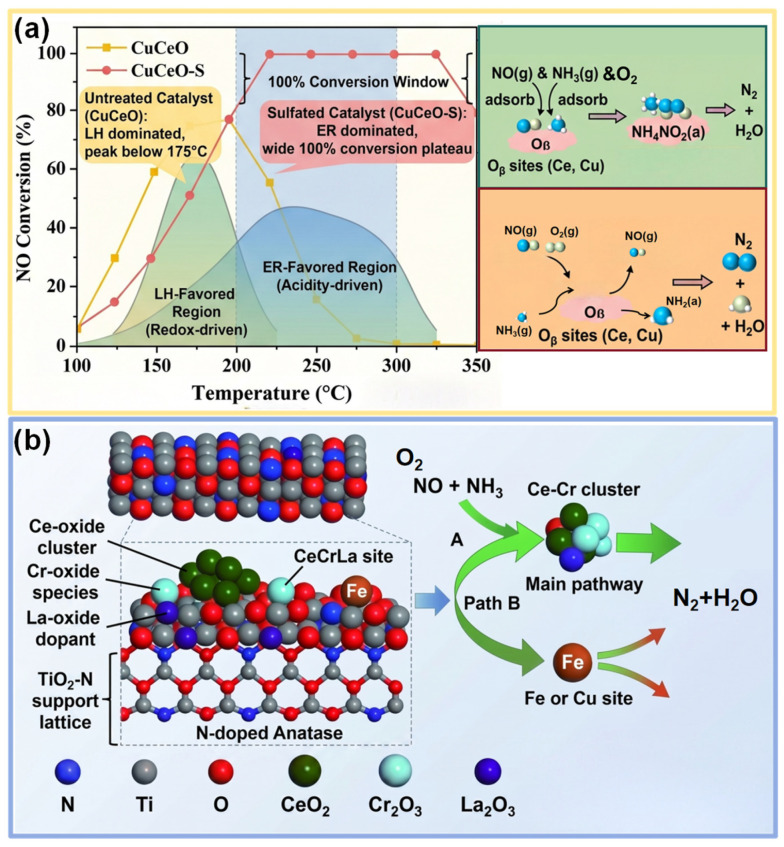
(**a**) schematic diagram of NO conversion and reaction mechanism of sulfuric acid-impregnated CuCeO catalyst, arrows represent the adsorption and conversion pathways of reactants and intermediates in L-H and E-R routes [121], (**b**) schematic diagram of structure and catalytic performance of N-doped CeCrLa/TiO_2_ catalyst, green arrows denote two parallel reaction pathways (Path A and Path B) toward final products N_2_ and H_2_O [122].

On this basis, in situ sulfur doping via precursors further achieves precise matching of acidity and redox capability. Different from conventional gas-phase sulfidation, this strategy employs organosulfur or sulfuric acid precursors to construct polymeric sulfate species on the catalyst surface, realizing charge redistribution of Ce-O bonds and targeted enhancement of acidity. Huang et al. prepared CeO_2_-TDC (TDC=2,5-thiophenedicarboxylic acid) using thiophenedicarboxylic acid as a precursor, which boosted the denitrification efficiency at 250 °C from 40% to 100% and exhibited excellent sulfur resistance. Liu et al. modified CuCeO via sulfuric acid impregnation to further balance low-temperature activity and wide-temperature applicability: the unmodified catalyst relied on redox performance at temperatures below 200 °C, whereas CuCeO-S, through the enhancement of Brønsted acid sites and activation of the Cu^2+^ + Ce^3+^↔Cu^+^ + Ce^4+^ redox cycle, achieved 100% NO conversion and N_2_ selectivity at 225–325 °C. This enables the reaction to proceed via both E-R and L-H mechanisms, greatly broadening the stable operating window under high-sulfur conditions (as shown in Figure 10a), arrows in the embedded mechanism insets represent the adsorption of gaseous reactants and the sequential transformation of intermediates on O_β_ sites; the upper pathway follows the E-R mechanism, while the lower pathway proceeds via the L-H mechanism [120,121].

#### 4.3.2. Nitrogen Doping Modification

Sulfur doping solves the problem of high-sulfur poisoning, but in sintering flue gas with high humidity, easy crystal phase transformation and complex poisons, the catalysts still face severe water inhibition, unstable supports and insufficient acidity matching. Nitrogen doping precisely targets these shortcomings: leveraging the unique electron-donating and structure-modifying properties of N atoms, it achieves synergistic enhancement in multiple dimensions, including crystal phase stabilization, active component dispersion improvement, electronic regulation and acidity optimization. Nitrogen doping is mainly realized through two approaches: support compounding and in situ doping, which improves the dispersion of active components such as Ce, Cr and La, and directionally regulates the ratio of redox couples (e.g., Cr^6+^/Cr and Ce^3+^/Ce), enhancing the content of surface adsorbed oxygen and oxygen migration ability. Meanwhile, nitrogen doping significantly strengthens Lewis acidity, making the reaction more inclined to follow the L-H mechanism and achieving nearly 100% NO*_x_* conversion in the ultra-wide-temperature range of 220–460 °C [122].

To further improve adaptability to low-temperature and multi-poison working conditions, researchers have developed advanced modification strategies combining composite nitrogen sources and atmosphere activation. Jiang et al. coupled n-butylamine with NH_3_ activation to induce crystal phase reconstruction and defect enrichment, increasing the density of oxygen vacancies and surface acid sites, accelerating the conversion of adsorbed NH_3_ and NO_2_ intermediates, and reducing carbon deposition and poison accumulation. This enabled the catalyst to maintain high efficiency and stability at 200–270 °C and exhibit excellent anti-poisoning performance in the coexistence of SO_2_ and H_2_O [123].

The CeCrLa/TiO_2_-N system using urea as the nitrogen source further realizes precise lattice-level regulation: N atoms stabilize the support structure through bonding modes such as O-Ti-N, induce the reduction in Ti^4+^ to Ti^3+^ and the enrichment of defect sites, and simultaneously improve the dispersion of active components and surface acidity. This system maintains high-efficiency denitrification in a wide-temperature range, with significantly enhanced structural stability, water resistance and anti-poisoning properties, providing a more reliable nitrogen doping scheme for high-humidity complex flue gas (as shown in Figure 10b), green arrows denote two parallel reaction pathways: Path A corresponds to the dominant main reaction route over Ce–Cr–La clusters, and Path B represents the secondary pathway mediated by Fe or Cu sites, both yielding final products N_2_ and H_2_O [122].

#### 4.3.3. Phosphorus Doping Modification

Catalyst sintering, pore collapse and active component agglomeration under long-term high-temperature operation cause efficiency degradation, which cannot be thoroughly solved by metal or single non-metal doping. Phosphorus doping, with its unique structural stabilization and acidity regulation capabilities, is a critical strategy to improve the anti-sintering property, sulfur resistance and long-cycle stability of cerium-based catalysts [124]. Its modification effect presents obvious concentration-dependent dual characteristics, which has become a research hotspot in recent years. For low-content phosphorus doping (≤5 wt%), PO_4_^3−^ species are uniformly distributed on the catalyst surface, which significantly increases the number of Brønsted acid sites and balances the redox performance. The optimized catalyst can maintain over 90% NO*_x_* conversion in the range of 240–420 °C. Excess phosphorus (≥10 wt%) will form massive stable CePO_4_, occupy active sites and destroy the Ce^3+^/Ce^4+^ redox cycle, leading to a sharp decline in catalytic activity [125]. For example, Zeng et al. prepared a phosphorus-modified CeTi catalyst. Through phosphorus doping, the specific surface area was increased from 35 m^2^/g to 160 m^2^/g, and the Ce^3+^ ratio was raised from 15.1% to 44.8%, with a NO conversion rate of up to 98% at 240 °C, far higher than that of the undoped sample [126].

#### 4.3.4. Boron (B) Doping Modification

As a typical non-metallic dopant, boron mainly exists in the form of B^3+^ in the ceria lattice. It has a small ionic radius and strong electron-withdrawing ability, which can effectively regulate the lattice structure, electronic distribution and surface defect sites of Ce-based catalysts, and is widely used to improve sulfur resistance and redox performance [127]. B^3+^ partially replaces Ce^4+^ in the CeO_2_ lattice, causing mild lattice distortion and inducing a large number of oxygen vacancies. Meanwhile, the electron-withdrawing effect of boron changes the electron density around Ce atoms: it reduces the surface electron density of Ce active sites and inhibits the adsorption and oxidation of SO_2_, so as to alleviate sulfate poisoning. Different from other dopants, boron doping will not excessively consume surface acid sites and can maintain the original NH_3_ adsorption capacity while improving anti-sulfur performance. DFT calculations by overseas teams verified that boron doping can increase the adsorption energy of SO_2_ on the catalyst surface by 0.4–0.6 eV, making SO_2_ difficult to react with Ce active components. Experimental results show that the optimal boron doping content is 2–4 wt%. The B-CeO_2_ catalyst maintains more than 88% NO conversion in the temperature range of 230–380 °C. Under the long-term atmosphere of 200 ppm SO_2_ and 8 vol% H_2_O, its activity only decreases by 11% after 40 h of continuous operation, which is far better than pure CeO_2_ [128].

#### 4.3.5. Fluorine (F) Doping Modification

Fluorine is a common anionic non-metallic dopant. F^−^ replaces lattice oxygen in CeO_2_, which has a significant regulatory effect on catalyst morphology, oxygen vacancy concentration and surface acid-base properties, and is an effective means to improve alkali resistance and hydrothermal stability of cerium-based catalysts [129].

After F^−^ replaces lattice oxygen, it will generate additional oxygen vacancies to maintain lattice charge balance. At the same time, fluorine doping can optimize the microscopic morphology of CeO_2_, transform stacked nanoparticles into dispersed 3D island structures, increase the specific surface area and expose more active sites. In terms of surface properties, F^−^ can regulate the ratio of Lewis and Brønsted acid sites, weaken the strong interaction between alkali metal ions and Ce active centers, and realize the targeted improvement of anti-alkali poisoning performance. In addition, the strong Ce-F bond enhances the lattice stability of the catalyst and inhibits crystal phase transformation under high humidity and high temperature [130].

To intuitively compare the comprehensive performance of different non-metal doped cerium-based catalysts, the key parameters, including active temperature window, denitrification efficiency and anti-poisoning capacity, are summarized in Table 2.

### 4.4. Support Modification Technology

Pure ceria-based catalysts are prone to agglomeration of active components, insufficient mass transfer efficiency and structural instability in industrial flue gas. Support modification is a modification technique that loads ceria-based active components onto the surface of functional supports with high specific surface area, well-developed pore structure and chemical stability. Supports can achieve high dispersion of active nanoparticles, construct unobstructed mass transfer channels for reactants, and also generate interfacial synergistic effects with ceria active sites, comprehensively improving the catalytic activity, poisoning resistance and sintering resistance of the catalysts. According to their material composition, mainstream supports are divided into three categories: porous molecular sieves, metal oxides and carbon materials.

#### 4.4.1. Porous Molecular Sieve Supports

Molecular sieves (SAPO-34, ZSM-5, SSZ-13, etc.) have regular microporous structures, large specific surface area and shape-selective effect, which are the most widely used supports in SCR catalysts. The pore confinement effect of molecular sieves can isolate large-particle toxic substances such as heavy metal oxides and ammonium salts, and protect internal Ce active sites. Overseas teams from the United States and Belgium have conducted in-depth research on molecular sieve-supported Ce-based catalysts: their experiments proved that SSZ-13-supported Ce catalysts can reduce the coverage rate of Pb and Cd pollutants by more than 40% through pore confinement [131].

In practical research, Ce/SSZ-13 and Ce/SAPO-34 are classic systems. The hierarchical pore structure of modified molecular sieves further optimizes the diffusion of reactants. After long-term operation in flue gas containing SO_2_ and K, the molecular sieve-supported catalyst still maintains 82% of the initial activity, while the pure CeO_2_ catalyst is almost completely deactivated. The main deficiency of molecular sieve supports is that they are prone to dealumination and pore collapse under high-temperature and high-humidity conditions, which limits their service life.

#### 4.4.2. Metal Oxide Supports

Common metal oxide supports include TiO_2_, Al_2_O_3_, ZrO_2_, etc. These supports have strong chemical stability and good interfacial interaction with CeO_2_. TiO_2_ is the mainstream support for industrial SCR catalysts: the strong metal-support interaction (SMSI) between TiO_2_ and Ce can regulate the electronic state of Ce and enhance redox capacity. ZrO_2_-doped TiO_2_ supports can further improve anti-sintering performance. European scholars found that Zr-Ti composite-supported Ce catalysts can maintain a stable pore structure after aging at 600 °C for 100 h.

Al_2_O_3_ has an ultra-high specific surface area, but its surface acid sites are easily neutralized by alkali metals. Therefore, surface coating modification is usually required when using Al_2_O_3_ as a support to improve alkali resistance. Metal oxide supports are suitable for medium-high temperature denitrification scenarios, and their low-temperature mass transfer efficiency needs to be further optimized.

#### 4.4.3. Carbon-Based Supports

Carbon materials (carbon nanotubes, activated carbon, graphene, etc.) have ultra-large specific surface area, flexible pore structure and excellent electrical conductivity. Carbon-based supports can promote electron migration between Ce active sites and significantly improve low-temperature activity. International research teams developed carbon nanotube (CNT) supported Ce catalysts, which achieve over 90% NO_x_ conversion at 100–200 °C [132].

However, carbon materials are easily oxidized and burned at temperatures above 400 °C, and are also prone to carbon deposition under the coexistence of VOCs. Therefore, carbon-based supported Ce catalysts are mostly applied to low-temperature flue gas denitrification below 350 °C.

### 4.5. Core-Shell Encapsulation Modification

Aiming at the problem that surface-active sites of Ce-based catalysts are directly eroded by various pollutants in industrial flue gas, core-shell encapsulation modification has become a frontier anti-poisoning technology. This structure takes Ce-based active components as the core, and coats a layer of inert or functional shell material on the surface. The shell acts as a physical barrier to block the contact between SO_2_, alkali metals and heavy metal pollutants and the core active sites; meanwhile, the interfacial effect between core and shell can further optimize the catalytic performance. According to shell materials, it is divided into oxide core-shell, molecular sieve core-shell, and carbon-based core-shell structures.

#### 4.5.1. Modification Mechanism of Core-Shell Structure

The core-shell structure, by constructing a functional partition design of “active core and protective shell”, has become an important structural regulation strategy for improving the poisoning resistance and structural stability of ceria-based catalysts. Its core functions are mainly reflected in three aspects: the dense protective shell can produce a physical isolation effect, effectively blocking toxic molecules from directly contacting the CeO_2_ active core and fundamentally preventing chemical poisoning and surface coverage of active sites; the porous shell possesses a molecular sieving effect that enables precise pore size regulation, allowing small molecule reactants such as NH_3_ and NO to freely penetrate and undergo catalytic reactions while intercepting large-particle poisons such as heavy metal agglomerates and ammonium salts; unique interfacial synergistic effects also occur at the core-shell interface, generating abundant additional oxygen vacancies and acid sites to assist the efficient progress of the SCR reaction, thereby effectively compensating for the slight activity loss caused by shell coating. A research team pioneered the systematic study on the anti-poisoning mechanism of oxide core-shell ceria-based catalysts, and experimental results confirmed that a rationally designed shell can reduce the sulfation reaction rate of CeO_2_ by more than 50%, fully verifying the great potential of the core-shell structure modification strategy in industrial flue gas denitrification applications [133].

#### 4.5.2. Typical Core-Shell Catalyst Systems

Based on different shell materials, core-shell ceria-based catalysts are mainly divided into three representative systems: oxide, molecular sieve and carbon-based core-shell structures. Among them, CeO_2_@TiO_2_ and CeO_2_@ZrO_2_ are the most typical oxide core-shell systems, where the TiO_2_ shell exhibits stable chemical properties and excellent sulfur resistance, enabling the CeO_2_@TiO_2_ catalyst to maintain NO*x* conversion above 85% after 50 h of operation in flue gas containing 300 ppm SO_2_ and showing far superior stability to uncoated CeO_2_. The molecular sieve core-shell structure, which features a Ce-based active core coated with a SAPO-34 or ZSM-5 molecular sieve shell, combines the shape-selective effect of molecular sieves with the high activity of Ce components, presenting outstanding resistance to alkali and heavy metal poisoning and being particularly suitable for high-alkali sintering flue gas. As for the carbon-based core-shell structure, CeO_2_@CNT core-shell catalysts are mainly applied in low-temperature denitrification, where the carbon shell significantly enhances electron transfer, allowing the catalyst to retain 80% of its initial activity after long-term exposure to Hg and Pb poisoning [134].

To fully evaluate the applicability of various modification routes for cerium-based catalysts, the core mechanism, advantages, limitations and applicable scenarios of all mainstream modification technologies are summarized in Table 3.

## 5. Conclusions

(1) Relying on the reversible Ce^3+^/Ce^4+^ redox cycle, high oxygen storage-release capacity, and tunable surface acidity, cerium-based catalysts have emerged as promising alternatives to conventional vanadium- and noble metal-based NH_3_-SCR denitrification catalysts, serving as environmentally benign core materials. They exhibit distinct merits, including low toxicity, minimal secondary pollution, and superior low-temperature adaptability, thereby demonstrating significant industrial application potential in the denitrification of industrial flue gas from steel sintering, hazardous waste incineration, and related processes.
(2) Complex industrial flue gas typically contains multiple impurities, including SO_2_, heavy metals, alkali/alkaline earth metals, H_2_O, P, HCl, and chlorobenzene, which induce catalyst deactivation via synergistic chemical poisoning and physical masking mechanisms. Moreover, the combined poisoning effect of multiple pollutants is substantially more severe than that of individual species, representing a critical bottleneck limiting practical engineering applications. Specifically, SO_2_ poisoning arises from the synergistic interaction between sulfate formation (chemical poisoning) and ammonium salt deposition (physical blockage). Heavy metals deactivate catalysts in a species-dependent manner by disrupting active sites, redox cycles, and surface structures. Alkali metals primarily deactivate catalysts through neutralization of surface acid sites and deterioration of redox properties. The inhibitory effect of H_2_O is strongly concentration-dependent, with high concentrations directly interrupting the electron transfer processes within the catalyst system.
(3) Elemental doping modification is widely recognized as an effective strategy to enhance the anti-poisoning performance of cerium-based catalysts. Rare earth doping can modulate the electronic structure, promote oxygen vacancy formation, optimize acid site distribution, and thereby improve both anti-poisoning resistance and structural stability. Transition metal (Mn, Fe, Cu) doping confers enhanced low-temperature activity, broadened resistance to sulphur and water over a wide-temperature range, and enables precise regulation of catalytic performance in the medium-to-low temperature window through synergistic effects. Non-metal (S, N, P) doping offers a novel pathway by transforming typical “poisons” (e.g., sulphur species) into active intermediates, stabilizing crystal structures, and suppressing high-temperature sintering. The integration of these three modification strategies enables the simultaneous enhancement of denitrification activity, anti-poisoning capability, and long-term operational stability.
(4) From the perspective of industrial flue gas governance, the optimized anti-poisoning cerium-based catalysts can fully adapt to the harsh working conditions of steel sintering flue gas with high pollutant concentration, fluctuating temperature and complex components. In compliance with China’s ultra-low emission standards for the iron and steel industry, this series of catalysts can stably control NO*x* emissions below 35 mg/m^3^, effectively solving the long-standing technical difficulties of denitrification for sintering flue gas. As a green alternative to traditional vanadium-based catalysts, it can eliminate the risk of heavy metal leakage and secondary pollution in industrial operations, and greatly reduce the operation and maintenance pressure of environmental protection facilities in iron and steel enterprises.

## 6. Prospects

(1) In terms of atmospheric environmental protection, the large-scale popularization of high-performance cerium-based SCR catalysts will significantly cut down NO_x_ emissions from key industrial sources. Nitrogen oxides are major precursors of haze, photochemical smog and acid rain. Efficient denitrification using cerium-based catalysts helps reduce regional air pollution, improve ambient air quality, and protect terrestrial and aquatic ecosystems.
(2) For low-carbon development and carbon neutrality goals, cerium-based catalysts have prominent advantages in the whole life cycle. Compared with traditional catalysts, their preparation process consumes less energy and produces fewer carbon emissions. Meanwhile, excellent low-temperature activity enables the denitrification system to operate at a lower temperature, reducing the heat supply demand of flue gas and cutting the energy consumption and carbon footprint of industrial enterprises. The popularization of this green catalytic technology can help traditional high-emission industries, such as iron and steel, realize energy saving, emission reduction and low-carbon transformation, and boost the implementation of national carbon peaking and carbon neutrality strategies.
(3) From the industrial and social benefits, China is rich in rare earth resources, and the industrialization of cerium-based denitrification catalysts can drive the high-value utilization of domestic rare earth resources, extend the rare earth industrial chain, and promote the upgrading of regional green environmental protection industries. In addition, the localization and popularization of low-cost, high-performance cerium-based catalysts can lower the threshold of industrial flue gas denitrification technology, enable more small and medium-sized industrial enterprises to meet ultra-low emission requirements, and promote the overall green and sustainable development of the manufacturing industry.

## Figures and Tables

**Table 1:** Comparison of catalytic performance loss, relative oxygen vacancy content and main deactivation modes of cerium-based catalysts under different flue gas poisoning conditions.

Poisoning Condition	Test Atmosphere and Duration	NO*_x_* Conversion Loss (%)	Relative Oxygen Vacancy Ratio (Fresh Catalyst = 100%)	Main Deactivation Mode	Ref.
Single SO_2_ poisoning	200 ppm SO_2_, 12 h	29	71	Chemical sulfation; active site occupation	[63]
Single H_2_O poisoning (8 vol%)	8 vol% H_2_O, 16 h	27	85	Competitive adsorption of reactants	[84]
Alkali metal (K) poisoning	K-containing flue gas, 20 h	~30	55	Surface acid neutralization; oxygen vacancy elimination	[78]
Heavy metal (Pb) poisoning	3 wt% Pb loading, 24 h	>30	62	Ce-O bond fracture; reaction pathway transformation	[97]

**Table 2:** Performance comparison of cerium-based catalysts modified by different non-metal elements (all performance data are obtained under simulated flue gas conditions with 100 ppm SO_2_ and 5 vol.% H2O; the test duration is 32 h for each group).

Doping Type	Optimal Temperature Range (°C)	Maximum NO_x_ Conversion (%)	Main Anti-Poisoning Performance	Core Technical Advantages	Ref.
Sulfur (S) doping	250~300	>90	Excellent SO_2_ resistance	Convert sulfur poisons into active sites; enrich Brønsted acid sites	[119,120]
Nitrogen (N) doping	220~460	Nearly 100	Good H_2_O and hydrothermal resistance	Inhibit TiO_2_ phase transformation; optimize surface acidity	[122]
Phosphorus (P) doping	240	98	High anti-sintering ability	Increase specific surface area; stabilize crystal structure	[126]
Boron (B) doping	230~380	>88	Superior long-term SO_2_ resistance	Regulate electronic structure; protect Ce active sites from sulfation	[128]
Fluorine (F) doping	210~450	Nearly 100	Outstanding alkali and hydrothermal resistance	Generate abundant oxygen vacancies; enhance lattice stability	[129]

**Table 3:** Summary of advantages, limitations and applicable conditions of different modification technologies for cerium-based catalysts.

Modification Technology	Core Modification Mechanism	Advantages	Limitations	Applicable Working Conditions	Ref.
Single rare earth doping	Regulate electronic structure; induce oxygen vacancies; optimize surface acid sites	Simple preparation; low cost; good structural stability	Limited comprehensive anti-poisoning performance; single regulation mode	Medium-temperature flue gas, low-pollution industrial scenarios	[103,104,105]
Transition metal doping (Mn/Fe/Cu)	Construct new redox cycles; optimize low-temperature reaction kinetics	Excellent low-temperature activity; wide active temperature window	Vulnerable to sulfur poisoning under long-term operation	Low-temperature industrial flue gas	[112,114,118]
Non-metal doping (S/N/P/F/B)	Transform poisons into active sites; enhance anti-sintering and acidity	Outstanding anti-sulfur and anti-sintering ability	Partial activity loss after long-term use	High-sulfur, high-temperature flue gas	[119,122,126,128,130]
Support modification	Disperse active components; build mass transfer channels; interfacial synergism	Improve anti-sintering and anti-blockage ability	support failure under extremely high-humidity conditions	Dust-containing industrial flue gas	[132]
Core-shell encapsulation	Physical isolation and molecular sieving; interfacial synergistic catalysis	Fundamental anti-poisoning effect; long service life	Complicated synthesis; high production cost; slight activity decline	Complex multi-pollutant flue gas (industrial sintering)	[133]

## Data Availability

No new data were created or analyzed in this study. Data sharing is not applicable to this article.
